# Peroxisome Proliferator Activated Receptor Ligands as Regulators of Airway Inflammation and Remodelling in Chronic Lung Disease

**DOI:** 10.1155/2007/14983

**Published:** 2007-08-20

**Authors:** Jane Elizabeth Ward, Xiahui Tan

**Affiliations:** Department of Pharmacology, University of Melbourne, Victoria 3010, Australia

## Abstract

Inflammation is a major component in the pathology of chronic lung diseases, including asthma. Anti-inflammatory treatment with corticosteroids is not effective in all patients. Thus, new therapeutic options are required to control diverse cellular functions that are currently not optimally targeted by these drugs in order to inhibit inflammation and its sequelae in lung disease. Peroxisome proliferator activated receptors (PPARs), originally characterised as regulators of lipid and glucose metabolism, offer marked potential in this respect. PPARs are expressed in both lung infiltrating and resident immune and inflammatory cells, as well as in resident and structural cells in the lungs, and play critical roles in the regulation of airway inflammation. In vitro, endogenous and synthetic ligands for PPARs regulate expression and release of proinflammatory cytokines and chemoattractants, and cell proliferation and survival. In murine models of allergen-induced inflammation, PPAR*α* and PPAR*γ* ligands reduce the influx of inflammatory cells, cytokine and mucus production, collagen deposition, and airways hyperresponsiveness. The activity profiles of PPAR ligands differ to corticosteroids, supporting the hypothesis that PPARs comprise additional therapeutic targets to mimimise the contribution of inflammation to airway remodelling and dysfunction.

## 1. INTRODUCTION

Current treatment for chronic lung diseases, including asthma, targets the inflammatory response that is a major contributor to disease pathology. Although inhaled corticosteroids are safe and effective in most patients, a significant proportion of patients with asthma fail to obtain the expected benefits of anti-inflammatory treatment or suffer adverse side effects, and these drugs have not been shown to prevent disease progression. The involvement of diverse cell types and mediators in the inflammatory process provides numerous potential therapeutic options in addition to those targeted by corticosteroids. Novel anti-inflammatory agents with different activity profiles to corticosteroids may minimise persistent inflammation and reduce its contribution to airway remodelling and airways hyperresponsiveness (AHR) in asthma and the loss of pulmonary function in other chronic inflammatory lung diseases.

Peroxisome proliferator activated receptors (PPARs) are ligand-activated transcription factors that have recently been implicated as targets for the regulation of inflammation. PPARs are members of the nuclear hormone receptor family with three isoforms, designated PPAR*α* (NR1C1), PPAR*β* (PPAR*δ*, NR1C2), and PPAR*γ* (NR1C3). Activation of these receptors has been shown to regulate diverse cellular responses including production of immunomodulatory cytokines, chemotaxis, cell differentiation, proliferation, and survival. This review describes the localisation of these receptors in key cells involved in the pathogenesis of inflammatory diseases in the lung, and presents in vitro and in vivo evidence describing the anti-inflammatory efficacy of PPAR ligands. The identification of complementary or additional actions to those exerted by corticosteroids supports further exploration of the therapeutic potential of PPAR ligands in asthma and chronic lung inflammation.

## 2. PPARs AND RXRs

The name PPAR derives from the identification of PPAR*α* as the molecular target for the fibrate class of drugs that induce peroxisome proliferation in rodents, a property not shared by the other PPAR isoforms. PPAR*α*, PPAR*β*, and PPAR*γ* share a common structure of 4 domains consisting of a variable amino terminal activation function-1 domain (AF-1, A/B), a DNA binding domain (C), a hinge region (D), and a highly conserved activation function-2 domain (AF-2, E/F). The large T-shaped ligand-binding domain within the AF-2 region enables PPARs to bind promiscuously to a plethora of structurally diverse endogenous and synthetic ligands [1]. In addition to ligand binding, AF-2 is important for association with coregulators of receptor activity, and for receptor dimerization and nuclear translocation. Unlike glucocorticoid receptors that form homodimers, PPARs exist as heterodimers with retinoid X receptors (RXR). Like PPARs, there are three distinct isoforms of RXR, namely RXR*α*, RXR*β*, and RXR*γ*, that are all activated by the endogenous ligand 9-cis retinoic acid [2].

## 3. PROPOSED MECHANISMS OF GENE REGULATION BY PPAR

The molecular mechanisms of gene regulation by PPARs are complex. Heterodimerization of PPAR with RXR may be affected by competition between PPAR isoforms and with other nuclear receptors that are also RXR partners, such as retinoic acid receptors, vitamin D receptors, and liver X receptors. In the absence of ligand, PPAR-RXR forms a complex with corepressor proteins with histone deacetylase activity, including nuclear receptor corepressor (NCoR) and the silencing mediator for retinoid and thyroid hormone receptors (SMRT) that prevents interaction with intracellular targets. Ligand binding causes corepressor dissociation, and ligand-dependent recruitment of coactivators such as steroid receptor coactivator-1 (SRC-1) and the PPAR binding protein (PBP) [3, 4].

Regulation of gene transcription can occur following nuclear translocation of this activated complex. Transcriptional activation or suppression can occur following recognition of PPAR response elements (PPRE) in promoters of target genes and binding to PPRE consensus sequences comprising AGGTCA hexamers separated by a single nucleotide spacer DR-1 (reviewed in [5]). Alternatively, PPAR can negatively regulate gene expression by antagonizing other signal-dependent transcription factors such as nuclear factor *κ*B (NF*κ*B), CAAT/enhancer binding protein (C/EBP), signal transducers and activators of transcription (STAT) or activator protein 1 (AP-1). This may occur via direct binding to cause transrepression [6] or by sequestering coactivators such as the glucocorticoid receptor interacting protein-1/transcriptional intermediary factor (GRIP-1/TIF) required for activity of other transcription factors [7]. PPAR*γ* ligands may also mediate responses via activation of mitogen-associated protein kinase (MAPK) and phosphoinositide-3-kinase (PI3K) pathways [8, 9]. The differential tissue distribution of PPAR*α*, PPAR*β*, and PPAR*γ* as well as competition between these isoforms and with other nuclear receptors for the accessory proteins that regulate their activity may allow the specific recognition of target genes and other transcription factors to modulate cell function [3].

## 4. PPAR TISSUE DISTRIBUTION AND LIGANDS

PPAR*α*, PPAR*β*, and PPAR*γ* are all widely expressed and share some common ligands. However, activation of a specific PPAR can be achieved using selective ligands in tissues where all isoforms are present or by targeting tissues where the isoforms are differentially expressed (Tables 1, 2).

PPAR*α* is highly expressed predominantly in liver, kidney, skeletal muscle, and heart, and has a role in the catabolism of fatty acids. Structurally diverse ligands for PPAR*α* include naturally occurring fatty acids and eicosanoids such as 8S-hydroxyeicosatetranoic acid (8S-HETE) and leukotriene B4 (LTB4). Among the synthetic PPAR*α* ligands described are the fibrate class of drugs used clinically for the treatment of dyslipidaemia such as clofibrate and fenofibrate, and pharmacological tools such as Wy14 643 [2].

The physiological role of PPAR*β* is less certain due to its ubiquitous expression. Fatty acids also activate PPAR*β*, with prostacyclin among other potential endogenous ligands for PPAR*β*. Recently developed synthetic ligands such as GW501516 and L165041 have been used to support a role for PPAR*β* in regulation of fatty acid oxidation and cell differentiation in skeletal muscle and adipose tissue [2, 4].

PPAR*γ* was originally characterised as a regulator of adipocyte differentiation, but also plays key roles in glucose and lipid metabolism. Activation of PPAR*γ* also occurs in response to a wide variety of potential endogenous ligands as well as synthetic agonists, such as the thiazolidinedione (TZD) class of insulin-sensitising drugs. Naturally occurring PPAR*γ* ligands include polyunsaturated fatty acids (PUFAs), such as linoleic acid, arachidonic acid, and eicosapentanoic acids, and oxidised lipids such as 9- and 13-hydroxyoctadecadienoic acid (HODE), and 12- and 15-HETE [10]. The arachidonic acid metabolite 15-deoxy-Δ^12, 14^-prostaglandin-J_2_ (15-d-PGJ_2_) has been widely used experimentally to define PPAR*γ*-dependent responses, despite additional actions mediated through PPAR*α* activation [11]. In this context, it is important to also note that it is still uncertain whether this prostaglandin D_2_ metabolite can be generated in vivo at the micromolar concentrations sufficient to mediate potential PPAR*γ*-dependent effects [12].

The TZD or glitazone class of drugs used in the treatment of type 2 diabetes are believed to exert their insulin-sensitising and hypoglycaemic effects through stimulation of PPAR*γ* [4]. Activation of PPAR*γ* by TZDs results in an alteration in the transcription of several genes involved in glucose and lipid utilisation such as GLUT4 glucose transporter and fatty acid transporter protein [13], and their binding affinity to PPAR*γ* closely parallels their in vivo hypoglycaemic potency [14]. These synthetic ligands include rosiglitazone (RGZ), ciglitazone (CGZ), pioglitazone (PGZ), and troglitazone (TGZ), with RGZ reported to be the most potent [15].

## 5. PPAR EXPRESSION AND ITS REGULATION IN INFLAMMATORY CELLS AND IN THE LUNG

Recent evidence supports a role for PPARs in the regulation of lung inflammation. Differential expression of PPAR isoforms has been demonstrated in different inflammatory, resident, and structural cells in the airways (see Table 2). Both PPAR*α* and PPAR*γ* are expressed in macrophages and monocytes [16–18], eosinophils [19, 20], with PPAR*β* also expressed in neutrophils [21, 22]. Dendritic cells express PPAR*γ* but not PPAR*α* [14, 23], but both PPAR*α* and PPAR*γ* are expressed in B and T lymphocytes [24, 25]. PPAR*β* and PPAR*γ* but not PPAR*α* are expressed in mast cells [26], while all three isoforms have been detected in A549 and BEAS-2B airway epithelial cell lines [27–30]. Mesenchymal expression of PPARs has been demonstrated with PPAR*γ* detected in primary fibroblasts [31], and with PPAR*α* and PPAR*γ* but not PPAR*β* in airway smooth muscle [13, 32]. The variable patterns of expression of these PPARs in these diverse cell types suggest that receptor activation of different isoforms may specifically modulate both the production of mediators implicated in inflammation and the cellular responses that contribute to tissue remodelling and the development of AHR.

Emerging evidence suggests that PPAR receptor expression is altered in lung disease, with changes in PPAR*γ* levels being the most extensively studied. PPAR*γ* has been localised in mucosal eosinophils and macrophages, airway epithelium and smooth muscle in human airway biopsies, with increased expression in asthmatic patients compared with controls [13]. In addition, in murine models of allergen-induced inflammation, higher levels of PPAR*γ* were evident in total lung extracts [36, 37] and could be localised to airway epithelium and muscle cells, mast cells, and some inflammatory cells [38].

The stimulus for the increased PPAR*γ* levels detected in intact airways is unclear, as is the functional role of this increase. In vitro, PPAR*γ* is inducible by the inflammatory cytokine interleukin-4 (IL-4) in airway epithelial cells and macrophages [27, 39]. PPAR*γ* expression in monocytes is increased with macrophage differentiation and activation [16, 17] and in sensitised mast cells following antigen exposure [26]. Similar changes in the cellular environment in asthmatic airways may contribute to the elevation in PPAR*γ* levels. The lower levels of PPAR*γ* expression in glucocorticoid-treated asthmatics, compared with untreated asthmatics [13], suggest that increased PPAR*γ* expression may be a product of the inflammatory pathways sensitive to steroid therapy. The current hypothesis is that PPAR*γ* expression is upregulated in response to inflammatory cytokines to provide a negative feedback mechanism, whereby endogenous PPAR*γ* ligands could activate these receptors to limit the cellular inflammatory response in the airways. Increases in PPAR*α* and PPAR*β* during inflammation have not yet been described, although PPAR*β* expression in the lung has recently been shown to be increased in the lungs of streptozotocin-induced diabetic rats [40].

However, PPAR upregulation does not appear to be a generalised response to inflammation. PPAR*α* and PPAR*β* were expressed in peripheral blood lymphocytes, monocytes, and neutrophils healthy subjects and in patients with cystic fibrosis (CF). However, relatively lower levels and activity of PPAR*α*, and not PPAR*β*, were detected in the lymphocytes from CF patients [22]. The authors speculate that this may be associated with changes in levels of endogenous PPAR*α* ligands in CF, but that treatment with synthetic PPAR*α* ligands may increase receptor expression and activity to minimise the immune response [41]. Although ovalbumin sensitisation has been shown to increase PPAR*γ* expression in the lung [36–38], levels of PPAR*α* were decreased in inflamed lungs of allergen-exposed mice [42]. In addition, a recent study has shown that PPAR*γ* mRNA and protein are downregulated in alveolar macrophages following segmental allergen challenge in asthmatic patients, but not healthy controls [43]. It has been suggested that this downregulation could contribute to ongoing pulmonary inflammation, tissue injury, and loss of function. Alternatively, it could accompany the reduction or resolution of inflammation following activation by PPAR*γ* ligands since increases in PPAR*γ* expression induced in a murine model of asthma by allergen sensitisation were inhibited by administration of the synthetic ligand, CGZ [38].

These studies suggest a complex interaction between the initiation and resolution of the inflammatory process and changes in PPAR receptor expression that may be regulated by both inflammatory mediators and the levels of endogenous PPAR ligands. It is important therefore to define receptor-mediated responses at both the cellular level and in integrated animal models of disease to elucidate the role for PPARs in lung inflammation.

Against this translational background, the development of selective ligands for PPAR isoforms has been critical. However, there are marked differences between reported binding affinities and receptor activation potencies for PPAR ligands versus the concentrations required to elicit cellular effects. Multiple approaches are therefore required to support claims for PPAR-dependency of these actions. These include confirming receptor expression in cells of interest and the use of pharmacological antagonists. Several irreversible antagonists for PPAR*γ* have been described including bisphenol A diglycidyl ether (BADGE) [44], and GW9662 [45], although the utility of the former may be compromised by its partial agonist activity [46]. Antagonists for other PPAR isoforms have been described (see Table 1), but have not been utilised in studies outlined in this review.

More recently, molecular techniques have been used to characterise potential PPAR-mediated responses. Adenoviral constructs expressing a dominant negative PPAR*γ* gene that binds to the ligand and the PPRE on DNA but does not initiate transcription have been used in lung fibroblasts [31], while the effects of overexpression of functional PPAR*γ* have been assessed in murine models of asthma [36, 37, 47]. In vivo, transgenic approaches have also characterised the regulatory role of PPAR*α* in inflammation using PPAR*α*-deficient mice [19, 48]. A similar approach for PPAR*γ* is not possible, since complete elimination of this isoform results in embryonic lethality. However, heterozygous knockout mice (PPAR*γ* ^+/−^) have been generated [49] and used to implicate PPAR*γ* in mast cell proliferation [50]. More recently, a developmental study using mice with specific ablation of PPAR*γ* in the airway epithelium showed that these conditionally PPAR^−/−^-targeted mice had reduced collagen extracellular matrix (ECM) gene expression in the lung [51]. This suggests that PPAR*γ* has a role in the epithelial-mesenchymal interactions necessary for the establishment of normal lung structure [51]. The implications of this finding on inflammation in lung disease have yet to be explored.

## 6. PPAR FUNCTION IN INFLAMMATORY CELLS AND IN THE LUNG

There is now extensive evidence that PPAR ligands regulate inflammatory and immune processes mediated by cells in which one or more PPAR isoforms is expressed. Many of these actions are in common with corticosteroids, which have been shown to have inhibitory effects on T cell, eosinophil, neutrophil, mast cell/basophil, and macrophage function [52]. PPAR ligands have also been shown to affect cellular responses of resident and structural cells implicated in inflammation and tissue remodelling in chronic lung diseases. Most of these studies have focussed on PPAR*γ*, and some direct comparisons have been made with corticosteroids [32, 53]. In many cases it remains to be determined whether the effects of PPAR ligands are receptor-mediated, to clarify differences in the activities and mechanisms of action of putative endogenous ligands and synthetic agonists, and to determine their potential therapeutic advantages over corticosteroids.

A characteristic feature of airway inflammation in asthma is the predominance of Th2 lymphocytes and their products, which mediate inflammatory cell recruitment of mast cells, eosinophils, and lymphocytes and subsequent release of mediators from these cells. Both 15-d-PGJ_2_ and CGZ are reported to inhibit T cell proliferation [24, 54]. 15-d-PGJ_2_ but not CGZ or PPAR*α* agonists induced T cell apoptosis [55]. 15-d-PGJ_2_ also decreased production of both Th1 and Th2 type cytokines from T cells [56]. In addition, T cells obtained from sensitised mice treated with CGZ showed decreased IFN*γ*, IL-4, and IL-2 release when exposed to allergen [56]. However, 15-d-PGJ_2_ also caused a potentially proinflammatory induction of IL-8 gene expression in human T cells and macrophages via a MAPK and/or NF*κ*B-dependent signalling pathway [8, 57].

In activated monocytes, the PPAR*γ* ligands 15-d-PGJ_2_ and TGZ inhibited production of proinflammatory cytokines tumour necrosis factor *α* (TNF*α*), interleukin-1*β* (IL-1*β*) and IL-6 [58]. In contrast, natural and synthetic agonists for PPAR*α* were ineffective in these cells [58]. 15-d-PGJ_2_ and RGZ also reduced TNF*α* release and the expression of inducible nitric oxide synthase (iNOS) and matrix metalloproteinase (MMP)-9 in activated macrophages, in part by antagonising the activities of AP-1, STAT, and NF*κ*B [17, 33]. Both PPAR*α* and PPAR*γ* agonists induced macrophage apoptosis, in cells stimulated with TNF*α* and IFN*γ* [16].

### 6.1. Eosinophils

Eosinophils are elevated in the airways of asthmatics and can release inflammatory and cytotoxic mediators, cytokines, and growth factors that contribute to tissue remodelling and AHR. IL-5 and eotaxin-induced chemotaxis were reduced by PPAR*α*- and PPAR*γ*-selective ligands Wy14 643 and RGZ at micromolar concentrations, with the effect of RGZ prevented by the PPAR*γ* antagonist, GW9662 [19]. In contrast to these findings, it has recently been shown that both 15-d-PGJ_2_ and TGZ prime eotaxin-induced chemotaxis in the picomolar to low nanomolar concentration range, and that this effect is also prevented by GW9662 [59]. The possibility that endogenous ligands may have proinflammatory effects via PPAR*γ* at physiological concentrations, but that exogenous ligands may be negative immunomodulators at higher concentrations, will require further investigation. This explanation may also resolve discrepancies in other in vitro studies examining regulation of cytokine release and expression that ascribe both pro- and anti-inflammatory properties to PPAR*γ* ligands.

### 6.2. Mast cells

Mast cell infiltration of airway smooth muscle (ASM) in the airway wall is associated with impaired function in asthma [60]. In response to antigen stimulation, mast cells release stored mediators such as histamine, and produce arachidonic acid derivatives such as prostaglandins and leukotrienes, and cytokines such as TNF*α*, IL-4, and granulocyte macrophage-colony stimulating factor (GM-CSF). Several roles for PPARs in regulation of mast cell function have been proposed. The PPAR*β* ligand carbaprostacyclin and PPAR*γ* ligands 15-d-PGJ_2_ and TGZ suppressed histamine release and TNF*α* and GM-CSF production by human basophilic KU812 cells and mast cells [26, 61]. In addition, the increase in cell surface expression of the high affinity IgE receptor Fc*ε*RI in response to antigen was reduced by selective PPAR*β* and PPAR*γ* ligands, namely PGA_1_ and 15-d-PGJ_2_ [61]. PPAR*α* ligands had no effect on cytokine release or Fc*ε*RI expression in these human cells [26, 61]. Although fenofibrate, Wy14 643, and CGZ inhibited antigen-induced leukotriene production from rat basophilic leukemia (RBL)-2H3 cells, these effects are likely to be PPAR-independent since PPAR*α* mRNA was below detection [34] and the inhibition by CGZ was not prevented by a PPAR*γ* antagonist or associated with nuclear translocation of the receptor [62]. However, proliferation of bone-marrow derived murine mast cells was increased by RGZ in an apparently PPAR*γ*-dependent manner, since it was prevented by the PPAR*γ* antagonist GW9662 and the effect of RGZ was reduced in cells from PPAR*γ* ^+/−^ mice [50].

Further studies are required to explore the mechanisms of action of these selective PPAR*β* and PPAR*γ* ligands, and to clarify the role of both receptors in the modulation of mast cell function. Previous in vivo studies suggest that corticosteroids have a minimal effect on mast cell degranulation and the appearance of mast cell mediators after segmental antigen challenge in subjects with asthma [63].

### 6.3. Epithelial cells

Epithelial remodelling has been documented early in the development of asthma. Airway epithelial cells can contribute to persistent inflammation through synthesis and secretion of enzymes and mediators that regulate matrix turnover and inflammatory cell influx. Potential anti-inflammatory actions for PPAR*γ* ligands have been described in various epithelial cell lines. RGZ and PGZ decreased TNF*α*- and phorbol myristate acetate (PMA)-induced MMP-9 activity levels in NL20 and BEAS cells, associated with inhibition of NF*κ*B activation [30]. In A549 cells, TZDs reduced expression of iNOS [27] and decreased secretion of Regulated upon Activation, Normal T-cell Expressed, and Secreted (RANTES) and IL-8 [27, 64]. In contrast to these studies, both RGZ and TGZ potentiated TNF*α*-induced production of proinflammatory cytokines GM-CSF, IL-6 and IL-8 from A549 cells, independently of PPAR*γ*, NF*κ*B or MAPK activation [65].

There is also a complex relationship between PPAR*γ* and cyclo-oxygenase-2 (COX-2) expression in airway epithelial cells. Both RGZ and CGZ inhibited increases in PMA-induced COX-2 expression by inhibiting AP-1 signalling [66]. However, TGZ increased basal and TNF*α*-induced COX-2 expression independently of PPAR*γ* and NF*κ*B, but dependent on PI3K and ERK MAPK pathways in A549 cells [9]. In this study, neither 15-d-PGJ_2_ nor RGZ (PPAR*γ* ligands), GW262570 (PPAR*γ*/*α* agonist), nor L-165041 (PPAR*β* agonist) regulated COX-2 expression. Further investigation of these discrepancies will be required to define the effects of PPAR*γ* ligands on airway epithelial cells and the mechanisms by which these pro- and/or anti-inflammatory responses occur.

### 6.4. Mesenchymal cells

Fibroblasts play an important role in regulation of ECM deposition that contributes to subepithelial fibrosis layer of the airway, and the development of fixed airway obstruction in asthma [67]. Thickening of the ASM layer is another characteristic feature of airway remodelling in asthma. This has been associated with ASM hypertrophy and/or hyperplasia [68, 69] and increased ECM deposition within the ASM bundle [70]. These cells can respond to both mitogens and inflammatory mediators and contribute to further changes in the airway through the production of ECM proteins, cytokines, and chemokines. The PPAR*γ* ligands RGZ, CGZ, and 15dPGJ_2_ inhibited the differentiation of human lung fibroblasts into myofibroblasts and reduce collagen I production following TGF*β* stimulation, although the PPAR*α* ligand Wy14 643 was ineffective [31]. These potential antifibrotic effects may be only partly mediated by PPAR*γ*, since they were suppressed by expression of a dominant negative PPAR*γ* construct, but not by GW9662 [31]. It has been reported that corticosteroids did not prevent TGF*β*-induced collagen I production by ASM cells from individuals with or without asthma [71], and this suggests that PPAR*γ* agonists may have a therapeutic advantage over corticosteroids in the regulation of lung fibrosis.

In ASM, the PPAR*γ* agonists 15-d-PGJ_2_ and CGZ suppressed both GM-CSF and G-CSF release [32, 53]. Interestingly, the profile of inflammatory mediator inhibition differed between CGZ and corticosteroids, as dexamethasone inhibited GM-CSF but not G-CSF levels [32, 53]. In a separate study, 15-d-PGJ_2_ and TGZ were shown to inhibit TNF*α*-induced production of eotaxin and monocyte chemotactic protein-1 (MCP-1) but not IL-8 secretion from ASM [53]. The inhibitory effects of 15-d-PGJ_2_ were additive with fluticasone, offering the intriguing possibility that PPAR*γ* agonists in combination with corticosteroids may provide additional therapeutic benefit in asthma [53]. Although PPAR*γ* heterodimerisation with the retinoid X receptor is well characterized, this study also described additional complexity in the mechanisms of action of PPAR*γ* agonists, with direct physical interactions between PPAR*γ* and GR [53]. In addition, PPAR*γ* agonists were shown to mediate anti-inflammatory effects directly via GR activation, with RGZ and CGZ stimulating GR nuclear translocation in a PPAR*γ* deficient cell line [72]. Assessment of these potential interactions between PPAR*γ* and GR by receptor translocation studies may provide additional insights into mechanisms underlying the relative activities of nuclear receptor agonists in ASM.

PPAR*γ* agonists CGZ and 15-d-PGJ_2_ increased COX-2 expression in ASM, by binding to the PPRE in the COX-2 promoter [35]. Despite the proposed proinflammatory role for this enzyme, the increased PGE_2_ levels following induction of COX-2 may act in an autocrine manner to reduce subsequent production of GM-CSF, and to inhibit proliferation of ASM [73, 74].

PPAR*γ* ligands may also exert direct antimitogenic actions that could inhibit airway remodelling. Both 15-d-PGJ_2_ and TZDs inhibited proliferation of human cultured ASM cells [32, 75]. The effects of 15-d-PGJ_2_ and RGZ were mitogen-independent, as each ligand decreased FGF2- and thrombin-mediated proliferation with similar potency [75]. However, only the effects of RGZ were reversed by the selective PPAR*γ* antagonist GW9662 [75]. RGZ caused inhibition of cell-cycle progression in late G1 phase, without decreasing mitogen-stimulated cyclin D1 protein levels, a mechanism that differs from dexamethasone [76]. Of additional interest, the degree of inhibition of serum-induced ASM proliferation was greater for CGZ than dexamethasone [32]. It is critical to extend these comparisons to cells derived from asthmatics where the ability of corticosteroids to inhibit proliferation is reduced [77] to determine whether PPAR*γ* provides an additional or alternative therapeutic target to glucocorticoid receptors to regulate remodelling in asthma.

PPAR*α* was also expressed in ASM, but surprisingly, Wy14 643 did not regulate inflammatory cytokine production [32, 53], COX-2 expression [35], or proliferation [32, 77] of these cells.

## 7. PPAR REGULATION OF ALLERGEN-INDUCED INFLAMMATION IN VIVO

On the basis of these in vitro findings in inflammatory and structural cells, the role of PPARs has now been explored in murine models of allergen-induced bronchial inflammation. Although these models do not recapitulate all the pathophysiological changes seen in asthma, sensitisation and repeated aerosol challenges with ovalbumin (OVA) induce airway eosinophilia, changes in airway structure and increases in airways reactivity. Numerous studies have now utilised synthetic PPAR ligands, adenoviral constructs carrying PPAR cDNA (AdPPAR) and transgenics to support immunomodulatory roles for PPARs in the regulation of inflammation, airway wall remodelling, and hyperresponsiveness. Although the majority of studies have focussed on PPAR*γ*, some comparisons have been made with the other PPAR isoforms.

## 7.1. PPARα

In PPAR*α*-deficient Balb/c mice sensitized and challenged with OVA, there were greater increases in lung inflammation, airway eosinophilia, and antigen-specific serum IgE levels than in wild-type OVA-treated mice [19]. This was associated with relatively higher IL-6, IL-13, and eotaxin levels in the lung, although IL-4, IL-5, and soluble vascular and cell adhesion molecule-1 (sVCAM-1) were not different [19]. Critically, the PPAR*α*-deficient Balb/c mice also displayed a greater response to MCh after OVA-sensitization and aerosol challenge than wild-type mice, providing a functional correlate to the cellular and humoral changes [19]. It was suggested that this PPAR*α* deletion worsened eosinophilia and asthma-like symptoms by preventing the anti-inflammatory actions mediated by the endogenous PPAR*α* ligand, LTB4, known to be produced abundantly by mast cells and other cell types in asthma. A separate study utilising a selective PPAR*α* ligand provides support for this explanation, since GW9578 inhibited allergen-induced influx of eosinophils and lymphocytes [28]. In the same study, the selective PPAR*β* agonist GW501516 had no effect on inflammatory cell influx [28].

There are conflicting reports on the role of PPAR*α* in regulating airway inflammation induced by lipopolysaccharide (LPS), which is characterised by infiltration of neutrophils and macrophages, increased chemoattractant levels, and elevated MMP activity in BALF. Although GW8578 had no effect on neutrophil influx or increased levels of keratinocyte derived-chemokine (KC) or TNF*α* following LPS treatment [28], fenofibrate reduced the increase in BALF neutrophils and macrophages as well as levels of TNF*α*, KC, macrophage inflammatory protein-2 (MIP-2), monocyte chemoattractant protein-1 (MCP-1) and both MMP-2 and MMP-9 activities [78]. Further studies are required to confirm if PPAR*α* activation may also have a beneficial effect in acute or chronic inflammatory airway disorders involving neutrophils and macrophages.

## 7.2. PPARγ

### 7.2.1. Inflammation

**Figure 1:**
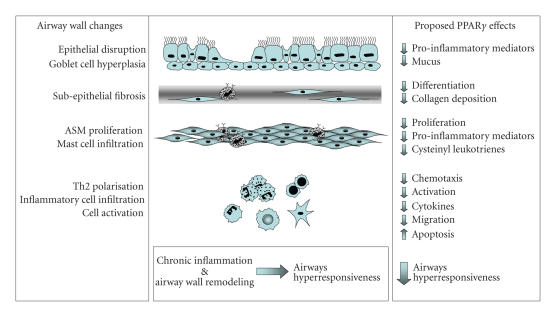
Proposed effects of PPAR*γ* ligands on inflammatory and remodelling changes in the asthmatic airway that contribute to airways hyperresponsiveness.

In Balb/c mice sensitized and challenged with OVA, PPAR*γ* ligands reduced levels of proinflammatory mediators in the bronchoalveolar lavage (BAL) fluid and lung [19, 28, 36, 37, 56] (see Figure 1). Significantly, cytokines associated with Th2-driven humoral responses were decreased by treatment with synthetic PPAR*γ* ligands. Increased levels of IL-4, IL-5, IL-13, and eosinophil cationic protein were reduced by the administration of RGZ or PGZ [36, 37]. In addition, in vitro studies of T cell obtained from sensitised mice treated with CGZ showed decreased IL-4 release when exposed to allergen [56].

Although the antigen sensitisation protocol differed, a common finding in these studies in Balb/c mice has been that PPAR*γ* ligands CGZ, RGZ and PGZ, and administration of AdPPAR*γ* reduced the OVA-induced influx of inflammatory cells, specifically eosinophils [19, 28, 36–38, 47] (see Figure 1). However, in C57BL/6 mice, RGZ treatment had no effect on levels of inflammatory cells in the bronchoalveolar lavage fluid despite inhibiting airways hyperresponsiveness [79]. Whether this is a strain difference in sensitivity to regulation of this measure of airway inflammation, or relates to the ligand and/or route of administration used is yet to be clarified.

### 7.2.2. Airway remodelling

Airway wall remodelling is characterised by goblet cell metaplasia, collagen deposition and subepithelial fibrosis, and smooth muscle hypertrophy and/or hyperplasia. The role of PPAR*γ* in regulating these changes has been explored. Treatment of Balb/c mice with nebulised CGZ was associated with a reduction in mucous production [38] (see Figure 1). Since orally administered CGZ had no effect [56] and oral RGZ treatment had no impact on goblet cell hyperplasia in C57BL/6 mice [79], the route of administration may be critical to regulate this parameter.

There is now evidence that activation of PPAR*γ* may regulate ECM deposition that occurs in airway wall remodelling. CGZ decreased basement membrane thickness and airways collagen deposition in response to antigen sensitization and challenge, associated with a reduction in TGF-*β* synthesis [38]. Inhibition of TGF-*β* signalling has also previously been shown to be inhibited by CGZ in cultured lung fibroblasts [31]. In support of a generalised antifibrotic activity for PPAR ligands, both RGZ and Wy14 643 have been shown to reduce bleomycin-induced pulmonary fibrosis in mice [18, 48, 80].

The antifibrotic effect of CGZ seen in vivo may also be related to regulation of the activity of MMPs or their inhibitors. Although an increase in MMP-2 proteolytic activity in the BALF with OVA treatment was not affected by RGZ [79], it has previously been reported that RGZ inhibits MMP-9 expression in bronchial epithelial cell lines [30].

Further studies are required to assess whether the inhibitory effects of TZDs on ASM proliferation translate to the in vivo setting, where antigen sensitization and challenge leads to inflammation, airway fibrosis, and thickening of the ASM layer. This is critical since the ability of corticosteroids to reduce airway structural changes in murine models is variable [81, 82], and airway remodelling persists despite optimal clinical use of corticosteroids in asthmatic patients [83].

### 7.2.3. Airways hyperresponsiveness

The impact of regulation of these markers of inflammation and tissue remodelling by PPAR*γ* ligands on AHR has also been explored. Using unrestrained plethysmography, the methacholine (MCh)-induced increase in enhanced pause (Penh) has been used as a measure of AHR following allergen sensitisation and challenge. In Balb/c mice, nebulized CGZ, oral RGZ, or oral PGZ completely prevented the increased response to MCh [19, 36–38, 47]. The effects of the synthetic PPAR*γ* ligands were mimicked by AdPPAR*γ* [36, 37, 47] and abrogated by GW9662 [19, 36–38, 47].

Using invasive measurements of respiratory resistance and compliance, RGZ reduced the increase in airways resistance after OVA challenge in C57BL/6 mice, without affecting the decrease in lung compliance, reflecting an effect on the airways rather than the parenchyma of the lung [79]. This finding provides further support for the proposed role of PPAR*γ* in the regulation of AHR, and is important in light of recent criticism of the use of Penh measurements to draw conclusions about the effects of potential therapeutic agents on AHR [84]. However, since this inhibition by RGZ occurred in the absence of a significant effect on the OVA-induced increase in BAL inflammatory cells, MMP-2 activity, and goblet cell number, it is possible that RGZ may modulate AHR by a mechanism that is independent of inhibition of inflammatory cell recruitment to the airway [79]. Dissociation between inhibition of eosinophilia and reduction in AHR have previously been reported, although the dose of dexamethasone required to inhibit AHR was higher than that needed to inhibit eosinophilia in a murine model of allergic airway inflammation [85].

### 7.2.4. Potential mechanisms for decreased inflammation and AHR by PPARγ ligands

MCh reactivity was unchanged in saline-challenged mice after oral treatment with RGZ for 7 days [79], suggesting that exposure of ASM to RGZ does not directly inhibit contractile responsiveness. In addition, RGZ did not modulate basal airway tone, or the contraction in response to MCh or histamine in isolated guinea pig tracheal rings [79]. However, CGZ and RGZ have been shown to produce concentration-dependent relaxation smooth muscle of isolated mouse trachea [86]. This effect was not prevented by GW9662, but was inhibited by indomethacin, and suggested that TZDs could act independently of PPAR*γ* to inhibit PGE_2_ metabolism by 15-hydroxyprostaglandin dehydrogenase leading to a dilator response [86, 87].

A series of studies have shown that inhibitory effects of TZDs and AdPPAR*γ* on both eosinophilia and AHR were prevented by GW9662 and implicate increases in phosphatase and tensin homologue deleted on chromosome ten (PTEN) and IL-10 in the protective roles of PPAR*γ*-activation [36, 37, 47]. This is in agreement with a separate study, in which RGZ inhibited the migration of antigen-loaded dendritic cells in the mediastinal lymph nodes and increased IL-10 production [88].

## 8. SUMMARY

On the basis of these in vitro and in vivo findings, substantial evidence has emerged to provide proof-of-concept for the future clinical application of PPAR*γ* ligands to treat airway inflammation. Given their long record of use in type 2 diabetes, TZDs appear to be ideally placed for use in the treatment of chronic lung disease. Like corticosteroids, they appear to have broad anti-inflammatory effects and possess potential anti-remodelling efficacy on multiple cell types in the lung and in animal models of asthma. However, PPAR*γ* agonists including RGZ may offer additional therapeutical advantages to current treatment, if they can be proven to exert control over proinflammatory and proasthmatic pathways that are not susceptible to inhibition by corticosteroids in the clinical setting. Further comparative studies are required to explore these novel complementary or additional actions of PPAR*γ* agonists to those already identified.

The efficacy of TZDs in asthma has not yet been evaluated in human clinical studies, although there has been a case report describing the reduction in asthma symptoms in patients treated with PGZ for diabetes [89]. A limited trial examining the effect of RGZ on lung function in comparison with low dose inhaled corticosteroids in steroid naïve smokers with asthma is currently underway in the United Kingdom to determine whether targeting PPAR*γ* may offer therapeutic benefit in the future treatment of asthma and other inflammatory lung diseases.

## Figures and Tables

**Table 1:** Natural and synthetic ligands for PPAR isoforms.

Isoform	Natural ligands	Synthetic ligands	Antagonists

PPAR*α*	PGD_2_	Fibrates	
	PGI_2_	Wy14 643	MK866
	LTB_4_	GW9578, GW7647	GW6471
	8S-HETE	NSAIDs	

PPAR*β*	PGA_1_	GW501516, GW0742	Sulindac
	PGI_2_	L165041	

PPAR*γ*	PGD_2_, PGJ_2,_15-d-PGJ_2_	TZDs	BADGE
	9-HODE, 13-HODE	GW262570	GW9662
	12-HETE, 15-HETE	NSAIDs	T0070907

PPAR*α*, *γ*	—	GW2331	—
		Ragaglitazar	

PPAR*α*, *β*, *γ*	Saturated FAs	—	—
	PUFAs		

BADGE: bisphenol A diglycidyl ether; FA: fatty acid; HETE: hydroxyeicosatetranoic acid; HODE: hydroxyoctadecadienoic acid; NSAID: nonsteroidal anti-inflammatory drug; PUFA: polyunsaturated fatty acid; TZD: thiazolidinedione.

**Table 2:** PPAR isoforms in inflammatory cells and lung structural cells.

Cell type	PPAR*α*	PPAR*β*	PPAR*γ*

Macrophage/monocyte	✓ [16, 22]	✓ [22, 33]	✓ [13, 17, 18, 33]
	× [33]	—	× [22]
Eosinophil	✓ [19]	—	✓ [13, 19, 20]
Neutrophils	✓ [22]	✓ [21, 22]	✓ [21]
	—	—	× [22]
Lymphocytes	✓ [22, 24, 25]	✓ [22]	✓ [24, 25]
	—	—	× [22]
Dendritic cells	× [14, 23]	—	✓ [14, 23]
Mast cells	× [26, 34]	✓ [26]	✓ [26]
Epithelial cells	✓ [28]	✓ [28, 29]	✓ [13, 27, 28, 30]
Lung fibroblasts	—	—	✓ [13, 31]
Airway smooth muscle	✓ [32, 35]	× [32, 35]	✓ [13, 32, 35]

## ABBREVIATIONS

AHR: Airways hyperresponsiveness
AP-1: Activator protein-1
ASM: Airway smooth muscle
BADGE: Bisphenol A diglycidyl ether
BALF: Bronchoalveolar lavage fluid
C/EBP: CAAT/enhancer binding protein
CF: Cystic fibrosis
CGZ: Ciglitazone
COX-2: Cyclo-oxygenase-2
15-d-PGJ_2_: 15-deoxy-Δ^12,14^-prostaglandin J_2_
ECM: Extracellular matrix
ERK: Extracellular regulated kinase
FA: Fatty acid
FGF-2: Fibroblast growth factor-2
G-CSF: Granulocyte colony stimulating factor
GM-CSF: Granulocyte-macrophage colony stimulating factor
GR: Glucocorticoid receptor
GRIP-1: Glucocorticoid receptor interacting protein-1
HETE: Hydroxyeicosatetranoic acid
HODE: Hydroxyoctadecadienoic acid
IFN*γ*: Interferon*γ*
iNOS: Inducible nitric oxide synthase
KC: Keratinocyte-derived chemokine
LPS: Lipopolysaccharide
LTB4: Leukotriene B4
MAPK: Mitogen-associated protein kinase
MCh: Methacholine
MCP-1: Monocyte chemotactic protein-1
MIP-2: Macrophage inflammatory protein-2
MMP: Matrix metalloprotease
NF*κ*B: Nuclear factor *κ*B
NSAID: Nonsteroidal anti-inflammatory drug
OVA: Ovalbumin
PGZ: Pioglitazone
PI3K: Phosphoinositide-3-kinase
PMA: Phorbol myristate acetate
PPAR: Peroxisome proliferator activated receptor
PPRE: Peroxisome proliferator activated receptor response element
PUFA: Polyunsaturated fatty acid
PTEN: Phosphatase and tensin homologue deleted on chromosome ten
RANTES: Regulated upon activation normal T cell expressed and secreted
RGZ: Rosiglitazone
RXR: Retinoid X receptor
STAT: Signal transducers and activators of transcription
sVCAM-1: Soluble vascular and cell adhesion molecule-1
TGF*β*: Transforming growth factor*β*
TGZ: Troglitazone
TIF: Transcriptional intermediary factor
TNF*α*: Tumour necrosis factor*α*
TZD: Thiazolidinedione

## References

[1] Nolte RT, Wisely GB, Westin S (1998). Ligand binding and co-activator assembly of the peroxisome proliferator-activated receptor-*γ*. *Nature*.

[2] Moraes LA, Piqueras L, Bishop-Bailey D (2006). Peroxisome proliferator-activated receptors and inflammation. *Pharmacology and Therapeutics*.

[3] Tien ES, Hannon DB, Thompson JT, Vanden Heuvel JP (2006). Examination of ligand-dependent coactivator recruitment by peroxisome proliferator-activated receptor-*α* (PPAR*α*). *PPAR Research*.

[4] Berger J, Moller DE (2002). The mechanisms of action of PPARs. *Annual Review of Medicine*.

[5] Desvergne B, Wahli W (1999). Peroxisome proliferator-activated receptors: nuclear control of metabolism. *Endocrine Reviews*.

[6] Straus DS, Pascual G, Li M (2000). 15-deoxyΔ^12,14^-prostaglandin J_2_ inhibits multiple steps in the NF-*κ*B signaling pathway. *Proceedings of the National Academy of Sciences of the United States of America*.

[7] Gervois P, Vu-Dac N, Kleemann R (2001). Negative regulation of human fibrinogen gene expression by peroxisome proliferator-activated receptor *α* agonists via inhibition of CCAAT box/enhancer-binding protein *β*. *Journal of Biological Chemistry*.

[8] Harris SG, Smith RS, Phipps RP (2002). 15-deoxy-Δ^12,14^-PGJ_2_ induces IL-8 production in human T cells by a mitogen-activated protein kinase pathway. *Journal of Immunology*.

[9] Patel KM, Wright KL, Whittaker P, Chakravarty P, Watson ML, Ward SG (2005). Differential modulation of COX-2 expression in A549 airway epithelial cells by structurally 
distinct PPAR*γ* agonists: evidence for disparate functional effects which are independent of NF-*κ*B and PPAR*γ*. *Cellular Signalling*.

[10] Willson TM, Brown PJ, Sternbach DD, Henke BR (2000). The PPARs: from orphan receptors to drug discovery. *Journal of Medicinal Chemistry*.

[11] Forman BM, Tontonoz P, Chen J, Brun RP, Spiegelman BM, Evans RM (1995). 15-deoxy-Δ^12,14^-prostaglandin J_2_ is a ligand for the adipocyte determination factor PPAR*γ*. *Cell*.

[12] Bell-Parikh LC, Ide T, Lawson JA, McNamara P, Reilly M, FitzGerald GA (2003). Biosynthesis of 15-deoxy-Δ^12,14^-PGJ_2_ and the ligation of PPAR*γ*. *Journal of Clinical Investigation*.

[13] Benayoun L, Letuve S, Druilhe A (2001). Regulation of peroxisome proliferator-activated receptor *γ* expression in human asthmatic airways: relationship with proliferation, apoptosis, and airway remodeling. *American Journal of Respiratory and Critical Care Medicine*.

[14] Faveeuw C, Fougeray S, Angeli V (2000). Peroxisome proliferator-activated receptor *γ* activators inhibit interleukin-12 production in murine dendritic cells. *FEBS Letters*.

[15] Lehmann JM, Moore LB, Smith-Oliver TA, Wilkison WO, Willson TM, Kliewer SA (1995). An antidiabetic thiazolidinedione is a high affinity ligand for peroxisome proliferator-activated receptor *γ* (PPAR*γ*). *Journal of Biological Chemistry*.

[16] Chinetti G, Griglio S, Antonucci M (1998). Activation of proliferator-activated receptors *α* and *γ* induces apoptosis of human monocyte-derived macrophages. *Journal of Biological Chemistry*.

[17] Ricote M, Huang J, Fajas L (1998). Expression of the peroxisome proliferator-activated receptor *γ* (PPAR*γ*) in human atherosclerosis and regulation in macrophages by colony stimulating factors and oxidized low density lipoprotein. *Proceedings of the National Academy of Sciences of the United States of America*.

[18] Standiford TJ, Keshamouni VG, Reddy RC (2005). Peroxisome proliferator-activated receptor-*γ* as a regulator of lung inflammation and repair. *Proceedings of the American Thoracic Society*.

[19] Woerly G, Honda K, Loyens M (2003). Peroxisome proliferator-activated receptors *α* and *γ* down-regulate allergic inflammation and eosinophil activation. *Journal of Experimental Medicine*.

[20] Ueki S, Adachi T, Bourdeaux J (2003). Expression of PPAR*γ* in eosinophils and its functional role in survival and chemotaxis. *Immunology Letters*.

[21] Greene ME, Blumberg B, McBride OW (1995). Isolation of the human peroxisome proliferator activated receptor *γ* cDNA: expression in hematopoietic cells and chromosomal mapping. *Gene Expression*.

[22] Reynders V, Loitsch S, Steinhauer C, Wagner T, Steinhilber D, Bargon J (2006). Peroxisome proliferator-activated receptor *α* (PPAR*α*) down-regulation in cystic fibrosis lymphocytes. *Respiratory Research*.

[23] Gosset P, Charbonnier A-S, Delerive P (2001). Peroxisome proliferator-activated receptor *γ* activators affect the maturation of human monocyte-derived dendritic cells. *European Journal of Immunology*.

[24] Clark RB, Bishop-Bailey D, Estrada-Hernandez T, Hla T, Puddington L, Padula SJ (2000). The nuclear receptor PPAR*γ* and immunoregulation: PPAR*γ* mediates inhibition of helper T cell responses. *Journal of Immunology*.

[25] Jones DC, Ding X, Daynes RA (2002). Nuclear receptor peroxisome proliferator-activated receptor *α* (PPAR*α*) is expressed in resting murine lymphocytes. The PPAR*α* in T and B lymphocytes is both transactivation and transrepression
competent. *Journal of Biological Chemistry*.

[26] Sugiyama H, Nonaka T, Kishimoto T, Komoriya K, Tsuji K, Nakahata T (2000). Peroxisome proliferator-activated receptors are expressed in human cultured mast cells: a possible role of these receptors in negative regulation of mast cell activation. *European Journal of Immunology*.

[27] Wang ACC, Dai X, Luu B, Conrad DJ (2001). Peroxisome proliferator-activated receptor-*γ* regulates airway epithelial cell activation. *American Journal of Respiratory Cell and Molecular Biology*.

[28] Trifilieff A, Bench A, Hanley M, Bayley D, Campbell E, Whittaker P (2003). PPAR-*α* and -*γ* but not -*δ* agonists inhibit airway inflammation in a murine model of asthma: in vitro evidence for an NF-*κ*B-independent effect. *British Journal of Pharmacology*.

[29] Arnold R, König W (2006). Peroxisome-proliferator-activated receptor-*γ* agonists inhibit the release of proinflammatory cytokines from RSV-infected epithelial cells. *Virology*.

[30] Hetzel M, Walcher D, Grüb M, Bach H, Hombach V, Marx N (2003). Inhibition of MMP-9 expression by PPAR*γ* activators in human bronchial epithelial cells. *Thorax*.

[31] Burgess HA, Daugherty LE, Thatcher TH (2005). PPAR*γ* agonists inhibit TGF-*β* induced pulmonary myofibroblast differentiation and collagen production: implications for therapy of lung fibrosis. *American Journal of Physiology*.

[32] Patel HJ, Belvisi MG, Bishop-Bailey D, Yacoub MH, Mitchell JA (2003). Activation of peroxisome proliferator-activated receptors in human airway smooth muscle cells has a superior anti-inflammatory profile to corticosteroids: relevance for chronic obstructive pulmonary disease therapy. *Journal of Immunology*.

[33] Ricote M, Li AC, Willson TM, Kelly CJ, Glass CK (1998). The peroxisome proliferator-activated receptor-*γ* is a negative regulator of macrophage activation. *Nature*.

[34] Yamashita M (2007). Peroxisome proliferator-activated receptor *α*-independent effects of peroxisome proliferators on cysteinyl leukotriene production in mast cells. *European Journal of Pharmacology*.

[35] Pang L, Nie M, Corbett L, Knox AJ (2003). Cyclooxygenase-2 expression by nonsteroidal anti-inflammatory drugs in human airway smooth muscle cells: role of peroxisome proliferator-activated receptors. *Journal of Immunology*.

[36] Kim SR, Lee KS, Park HS (2005). Involvement of IL-10 in peroxisome proliferator-activated receptor *γ*-mediated anti-inflammatory response in asthma. *Molecular Pharmacology*.

[37] Lee KS, Park SJ, Hwang PH (2005). PPAR-*γ* modulates allergic inflammation through up-regulation of PTEN. *The FASEB Journal*.

[38] Honda K, Marquillies P, Capron M, Dombrowicz D (2004). Peroxisome proliferator-activated receptor *γ* is expressed in airways and inhibits features of airway remodeling in a mouse asthma model. *Journal of Allergy and Clinical Immunology*.

[39] Huang JT, Welch JS, Ricote M (1999). Interleukin-4-dependent production of PPAR-*γ* ligands in macrophages by 12/15-lipoxygenase. *Nature*.

[40] Huang C-J, Liu I-M, Cheng J-T (2007). Increase of peroxisome proliferator-activated receptor *δ* gene expression in the lungs of streptozotocin-induced diabetic rats. *Pulmonary Pharmacology & Therapeutics*.

[41] Inoue I, Shino K, Noji S, Awata T, Katayama S (1998). Expression of peroxisome proliferator-activated receptor *α* (PPAR*α*) in primary cultures of human vascular endothelial cells. *Biochemical and Biophysical Research Communications*.

[42] Becker J, Delayre-Orthez C, Frossard N, Pons F (2006). Regulation of inflammation by PPARs: a future approach to treat lung inflammatory diseases?. *Fundamental and Clinical Pharmacology*.

[43] Kobayashi M, Thomassen MJ, Rambasek T (2005). An inverse relationship between peroxisome proliferator-activated receptor *γ* and allergic airway inflammation in an allergen challenge model. *Annals of Allergy, Asthma & Immunology*.

[44] Wright HM, Clish CB, Mikami T (2000). A synthetic antagonist for the peroxisome proliferator-activated receptor *γ* inhibits adipocyte differentiation. *Journal of Biological Chemistry*.

[45] Leesnitzer LM, Parks DJ, Bledsoe RK (2002). Functional consequences of cysteine modification in the ligand binding sites of peroxisome proliferator activated receptors by GW9662. *Biochemistry*.

[46] Bishop-Bailey D, Hla T, Warner TD (2000). Bisphenol A diglycidyl ether (BADGE) is a PPAR*γ* agonist in an ECV304 cell line. *British Journal of Pharmacology*.

[47] Lee KS, Kim SR, Park SJ (2006). Peroxisome proliferator activated receptor-*γ* modulates reactive oxygen species generation and activation of nuclear factor-*κ*B and hypoxia-inducible factor 1*α* in allergic airway disease of mice. *Journal of Allergy and Clinical Immunology*.

[48] Genovese T, Mazzon E, Di Paola R (2005). Role of endogenous and exogenous ligands for the peroxisome proliferator-activated receptor *α* in the development of bleomycin-induced lung injury. *Shock*.

[49] Miles PDG, Barak Y, He W, Evans RM, Olefsky JM (2000). Improved insulin-sensitivity in mice heterozygous for PPAR-*γ* deficiency. *Journal of Clinical Investigation*.

[50] Maeyama K, Emi M, Tachibana M (2005). Nuclear receptors as targets for drug development: peroxisome proliferator-activated receptor *γ* in mast cells: its roles in proliferation and differentiation. *Journal of Pharmacological Sciences*.

[51] Simon DM, Arikan MC, Srisuma S (2006). Epithelial cell PPAR*γ* contributes to normal lung maturation. *The FASEB Journal*.

[52] Belvisi MG (2004). Regulation of inflammatory cell function by corticosteroids. *Proceedings of the American Thoracic Society*.

[53] Nie M, Corbett L, Knox AJ, Pang L (2005). Differential regulation of chemokine expression by peroxisome proliferator-activated receptor *γ* agonists: interactions with glucocorticoids and *β*
_2_-agonists. *Journal of Biological Chemistry*.

[54] Cunard R, Ricote M, DiCampli D (2002). Regulation of cytokine expression by ligands of peroxisome proliferator activated receptors. *Journal of Immunology*.

[55] Harris SG, Phipps RP (2002). Prostaglandin D_2_, its metabolite 15-d-PGJ_2_, and peroxisome proliferator activated receptor-*γ* agonists induce apoptosis in transformed, but not normal, human T lineage cells. *Immunology*.

[56] Mueller C, Weaver V, Vanden Heuvel JP, August A, Cantorna MT (2003). Peroxisome proliferator-activated receptor *γ* ligands attenuate immunological symptoms of experimental allergic asthma. *Archives of Biochemistry and Biophysics*.

[57] Fu Y, Luo N, Lopes-Virella MF (2002). Upregulation of interleukin-8 expression by prostaglandin D_2_ metabolite 15-deoxy-Δ^12,14^ prostaglandin J_2_ (15d-PGJ_2_) in human THP-1 macrophages. *Atherosclerosis*.

[58] Jiang C, Ting AT, Seed B (1998). PPAR-*γ* agonists inhibit production of monocyte inflammatory cytokines. *Nature*.

[59] Kobayashi Y, Ueki S, Mahemuti G (2005). Physiological levels of 15-deoxy-Δ^12,14^-prostaglandin J_2_ prime eotaxin-induced chemotaxis on human eosinophils through peroxisome proliferator-activated receptor-*γ* ligation. *Journal of Immunology*.

[60] Brightling CE, Bradding P, Symon FA, Holgate ST, Wardlaw AJ, Pavord ID (2002). Mast-cell infiltration of airway smooth muscle in asthma. *New England Journal of Medicine*.

[61] Fujimura Y, Tachibana H, Yamada K (2002). Peroxisome proliferator-activated receptor ligands negatively regulate the expression of the high-affinity IgE receptor Fc*ε*RI in human basophilic KU812 cells. *Biochemical and Biophysical Research Communications*.

[62] Okuyama K, Yamashita M, Kitabatake Y, Kawamura S, Takayanagi M, Ohno I (2005). Ciglitazone inhibits the antigen-induced leukotrienes production independently of PPAR*γ* in RBL-2H3 mast cells. *European Journal of Pharmacology*.

[63] Liu MC, Proud D, Lichtenstein LM (2001). Effects of prednisone on the cellular responses and release of cytokines and mediators after segmental allergen challenge of asthmatic subjects. *Journal of Allergy and Clinical Immunology*.

[64] Momoi A, Murao K, Imachi H (1999). Thiazolidinedione inhibits production of RANTES in a cytokine-treated human lung epithelial cell line. *FEBS Letters*.

[65] Desmet C, Warzée B, Gosset P (2005). Pro-inflammatory properties for thiazolidinediones. *Biochemical Pharmacology*.

[66] Subbaramaiah K, Lin DT, Hart JC, Dannenberg AJ (2001). Peroxisome proliferator-activated receptor *γ* ligands suppress the transcriptional activation of cyclooxygenase-2. Evidence for involvement of activator protein-1 and CREB-binding protein/p300. *Journal of Biological Chemistry*.

[67] Bousquet J, Chanez P, Lacoste JY (1991). Indirect evidence of bronchial inflammation assessed by titration of inflammatory mediators in BAL fluid of patients with asthma. *Journal of Allergy and Clinical Immunology*.

[68] Ebina M, Takahashi T, Chiba T, Motomiya M (1993). Cellular hypertrophy and hyperplasia of airway smooth muscles underlying bronchial asthma. A 3-D morphometric study. *American Review of Respiratory Disease*.

[69] Woodruff PG, Dolganov GM, Ferrando RE (2004). Hyperplasia of smooth muscle in mild to moderate asthma without changes in cell size or gene expression. *American Journal of Respiratory and Critical Care Medicine*.

[70] Thomson RJ, Bramley AM, Schellenberg RR (1996). Airway muscle stereology: implications for increased shortening in asthma. *American Journal of Respiratory and Critical Care Medicine*.

[71] Burgess JK, Oliver BGG, Poniris MH (2006). A phosphodiesterase 4 inhibitor inhibits matrix protein deposition in airways in vitro. *Journal of Allergy and Clinical Immunology*.

[72] Ialenti A, Grassia G, Di Meglio P, Maffia P, Di Rosa M, Ianaro A (2005). Mechanism of the anti-inflammatory effect of thiazolidinediones: relationship with the glucocorticoid pathway. *Molecular Pharmacology*.

[73] Saunders MA, Mitchell JA, Seldon PM (1997). Release of granulocyte-macrophage colony stimulating factor by human cultured airway smooth muscle cells: suppression by dexamethasone. *British Journal of Pharmacology*.

[74] Belvisi MG, Saunders M, Yacoub M, Mitchell JA (1998). Expression of cyclo-oxygenase-2 in human airway smooth muscle is associated with profound reductions in cell growth. *British Journal of Pharmacology*.

[75] Ward JE, Gould H, Harris T, Bonacci JV, Stewart AG (2004). PPAR*γ* ligands, 15-deoxy-Δ2,14-prostaglandin J_2_ and rosiglitazone regulate human cultured airway smooth muscle proliferation through different mechanisms. *British Journal of Pharmacology*.

[76] Fernandes D, Guida E, Koutsoubos V (1999). Glucocorticoids inhibit proliferation, cyclin D1 expression, and retinoblastoma protein phosphorylation, but not activity of the extracellular-regulated kinases in human cultured airway smooth muscle. *American Journal of Respiratory Cell and Molecular Biology*.

[77] Johnson PRA, Burgess JK, Underwood PA (2004). Extracellular matrix proteins modulate asthmatic airway smooth muscle cell proliferation via an autocrine mechanism. *Journal of Allergy and Clinical Immunology*.

[78] Delayre-Orthez C, Becker J, Guenon I (2005). PPAR*α* downregulates airway inflammation induced by lipopolysaccharide in the mouse. *Respiratory Research*.

[79] Ward JE, Fernandes DJ, Taylor CC, Bonacci JV, Quan L, Stewart AG (2006). The PPAR*γ* ligand, rosiglitazone, reduces airways hyperresponsiveness in a murine model of allergen-induced inflammation. *Pulmonary Pharmacology & Therapeutics*.

[80] Genovese T, Cuzzocrea S, Di Paola R (2005). Effect of rosiglitazone and 15-deoxy-Δ^12,14^-prostaglandin J_2_ on bleomycin-induced lung injury. *European Respiratory Journal*.

[81] Trifilieff A, El-Hashim A, Bertrand C (2000). Time course of inflammatory and remodeling events in a murine model of asthma: effect of steroid treatment. *American Journal of Physiology*.

[82] Lee SY, Kim JS, Lee JM Inhaled corticosteroid prevents the thickening of airway smooth muscle in murine model of chronic asthma.

[83] Ward C, Walters H (2005). Airway wall remodelling: the influence of corticosteroids. *Current Opinion in Allergy and Clinical Immunology*.

[84] Bates J, Irvin C, Brusasco V (2004). The use and misuse of Penh in animal models of lung disease. *American Journal of Respiratory Cell and Molecular Biology*.

[85] Birrell MA, Battram CH, Woodman P, McCluskie K, Belvisi MG (2003). Dissociation by steroids of eosinophilic inflammation from airway hyperresponsiveness in murine airways. *Respiratory Research*.

[86] Henry PJ, D'Aprile A, Self G, Hong T, Mann TS (2005). Inhibitors of prostaglandin transport and metabolism augment protease-activated receptor-2-mediated increases in prostaglandin E_2_ levels and smooth muscle relaxation in mouse isolated trachea. *Journal of Pharmacology and Experimental Therapeutics*.

[87] Cho H, Tai H-H (2002). Thiazolidinediones as a novel class of NAD^+^-dependent 15-hydroxyprostaglandin dehydrogenase inhibitors. *Archives of Biochemistry and Biophysics*.

[88] Hammad H, de Heer HJ, Soullié T (2004). Activation of peroxisome proliferator-activated receptor-*γ* in dendritic cells inhibits the development of eosinophilic airway inflammation in a mouse model of asthma. *American Journal of Pathology*.

[89] Hashimoto Y, Nakahara K (2002). Improvement of asthma after administration of pioglitazone. *Diabetes Care*.

